# Experimental challenge with bovine respiratory syncytial virus in dairy calves: bronchial lymph node transcriptome response

**DOI:** 10.1038/s41598-019-51094-z

**Published:** 2019-10-14

**Authors:** Dayle Johnston, Bernadette Earley, Matthew S. McCabe, Ken Lemon, Catherine Duffy, Michael McMenamy, S. Louise Cosby, JaeWoo Kim, Gordon Blackshields, Jeremy F. Taylor, Sinead M. Waters

**Affiliations:** 1 Animal and Bioscience Research Department, Animal & Grassland Research and Innovation Centre, Teagasc, Grange, Co. Meath Ireland; 2 Veterinary Sciences Division, Agri-Food and Biosciences Institute, Stormont, Belfast, Northern Ireland; 3 Division of Animal Sciences, University of Missouri, Columbia, MO USA

**Keywords:** Transcriptomics, Viral infection

## Abstract

Bovine Respiratory Disease (BRD) is the leading cause of mortality in calves. The objective of this study was to examine the response of the host’s bronchial lymph node transcriptome to Bovine Respiratory Syncytial Virus (BRSV) in a controlled viral challenge. Holstein-Friesian calves were either inoculated with virus (10^3.5^ TCID_50_/ml × 15 ml) (n = 12) or mock challenged with phosphate buffered saline (n = 6). Clinical signs were scored daily and blood was collected for haematology counts, until euthanasia at day 7 post-challenge. RNA was extracted and sequenced (75 bp paired-end) from bronchial lymph nodes. Sequence reads were aligned to the UMD3.1 bovine reference genome and differential gene expression analysis was performed using EdgeR. There was a clear separation between BRSV challenged and control calves based on gene expression changes, despite an observed mild clinical manifestation of the disease. Therefore, measuring host gene expression levels may be beneficial for the diagnosis of subclinical BRD. There were 934 differentially expressed genes (DEG) (p < 0.05, FDR <0.1, fold change >2) between the BRSV challenged and control calves. Over-represented gene ontology terms, pathways and molecular functions, among the DEG, were associated with immune responses. The top enriched pathways included interferon signaling, granzyme B signaling and pathogen pattern recognition receptors, which are responsible for the cytotoxic responses necessary to eliminate the virus.

## Introduction

Bovine Respiratory Disease (BRD) is the leading cause of morbidity and mortality in calves over one month of age in Ireland^1–4^ and internationally^5–8^. It is a multifactorial disease, involving infectious agents (both viral and bacterial), host factors, management practices, environmental stress factors and their interactions^4,8–11^. Viruses generally initiate BRD and the damage they cause to the respiratory tract predisposes the calves to a secondary infection caused by bacteria, many of which are normal flora of the bovine upper respiratory tract^12^.

Bovine Respiratory Syncytial Virus (BRSV) is one of the main viral infectious agents responsible for the onset of BRD^13^. BRSV is an enveloped, non-segmented, negative-stranded RNA virus and is a member of the *Orthopneumovirus* genus within the family *Pneumoviridae*^14,15^. The morbidity resulting from infection with BRSV is in the range of 60% to 80%, and mortality is reported to be 20%^15^. The disease resulting from infection with BRSV can vary from sub-clinical to severe manifestation of clinical signs including coughing, pyrexia, nasal discharge, anorexia and respiratory distress^15^. Lung pathology resulting from BRSV infection is primarily due to the host inflammatory response, particularly the induction of pro-inflammatory cytokines (IL12B, IFNG, TNF, IL6, IL18, CXCL8, CCL5, CCL2, CCL3, IFNA1 and IFNB1) and the influx of leukocytes, particularly neutrophils^13,15^. Establishment and maintenance of BRSV infection is facilitated through the virus’ ability to interfere with the host’s interferon (anti-viral) response and to induce immunomodulation by shifting the adaptive immune response away from a cell mediated response towards a Th2 dominated response^15,16^.

Despite the risk of BRD susceptibility being moderately heritable^17^, there is a paucity of literature describing the molecular immune response of the host to infection with agents of the bovine respiratory disease complex (BRDC), such as BRSV. Two RNA-Seq studies performed in crossbred Angus-Hereford beef calves that were artificially challenged with single pathogens of the BRDC at the University of California Davis have discovered multiple genes and pathways to be differentially expressed following a BRSV challenge in bronchial lymph node^18^ and in multiple lymphoid and lung tissues^19^. However, no work has been carried out to date to examine the transcriptional response to a BRSV challenge in artificially-reared Irish dairy bull calves. The aims of this study were: (1) to describe the clinical and haematological response to a BRSV challenge infection, (2) to elucidate the genes and pathways involved in the host’s bronchial lymph node transcriptome response to an experimental challenge with BRSV in Irish artificially-reared dairy calves, and (3) to compare the bronchial lymph node transcriptomic response of these dairy calves challenged with BRSV to that of the crossbred beef calves challenged with BRSV at the University of California Davis^18^. These differentially expressed genes (DEG), and in particular the genes which were commonly differentially expressed in both the present study and the US study^18^ may harbour variants which influence the host’s resistance to BRSV.

## Materials and Methods

### Preparation of the BRSV Inoculum

Foetal calf lung (FCL) primary cells were grown in T75 tissue culture flasks using G-MEM BHK-21 media (Gibco #21710-025) with 10% foetal calf serum. BRSV strain SVA 274/92 cell lysate^20^ was removed from the −80 °C freezer and defrosted. A 1:7 dilution was prepared using G-MEM BHK-21 media containing 2% foetal calf serum. Growth media was removed and the virus stock (2 ml) was added to each flask of FCL cells and incubated at 37^o^C in 5% CO_2_ for 1 hour. The flasks were removed from the incubator and G-MEM BHK-21 media (18 ml) containing 2% foetal calf serum was added. The flasks were transferred to the incubator and incubated until the cytopathic effect was greater than 50%. Cell sheets were freeze-thawed to release intracellular virus, centrifuged at 500 g for 5 minutes at 4 °C to remove cellular debris, aliquoted and stored at −80 °C.

### Animal model

All animal studies were carried out in accordance with the UK Animals (Scientific Procedures) Act 1986 and with the approval of the Agri-Food and Biosciences Institute Northern Ireland Ethical Review Committee.

Animals were selected from a population of 30 Holstein-Friesian bull calves (mean age ± s.d. = 120.7 ± 14.15 days) and recruited into the study based on low BRSV specific maternally derived antibody (MDA) levels and negative BRSV PCR status two weeks prior to challenge. As the challenge and necropsy were staggered across three days, recruited animals were assigned to three groups (A, B and C) based on sire, age (A = 135 ± 14 days, B = 134 ± 16 days, C = 133 ± 22 days), weight (A = 154.7 ± 14.0 kg, B = 155.0 ± 14.00 kg, C = 155 ± 19.00 kg) and MDA levels (A = 11.9 ± 5.48 MDA, B = 11.8 ± 5.15 MDA, C = 11.3 ± 6.13 MDA). The study included two treatments; calves were either challenged with BRSV (n = 12; groups B and C) or were mock challenged with sterile phosphate buffered saline (PBS) (n = 6; group A) (Control). The difference between treatment groups B and C was that they were housed in separate locations.

### Animal accommodation

**Figure 1:**
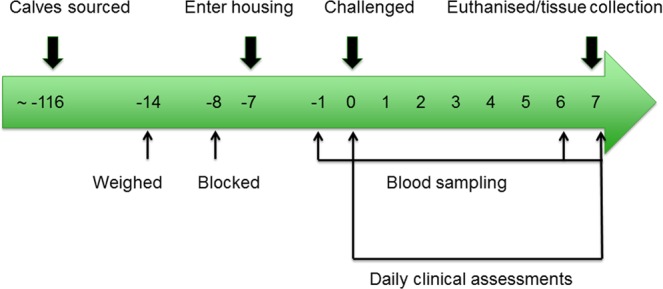
The BRSV challenge timeline. The numbers within the timeline represent the number of days relative to the day of the challenge (day 0).

The calves entered and were acclimatised to their houses on day −7 relative to the challenge (Fig. 1). Two of the houses (housing groups A and B) were class 3 animal houses which were identical in layout (10.03 m × 5.01 m) while the third house was an older, separate house (6.65 m × 3.70 m). The floors were covered with sawdust and a calf restraining chute was contained within each of the calf houses.

### Animal diet

Prior to the trial, from arrival to Agri-Food Biosciences Institute (AFBI) Stormont at 3 weeks of age, the calves were reared indoors and received 2 feeds per day of 2 litres of 23% protein, 23% fat, calf milk replacer (Thompsons high fat; Trouw Nutrition Limited), *ad libitum* silage and approximately 200 g of calf weaner nuts (Calf Pride Weaner Mix; John Thompson and Sons, Limited) to encourage them to start eating concentrates. They were weaned from the calf milk replacer at 8–10 weeks of age and subsequently fed *ad libitum* silage and approximately 1.5 kg of Calf Pride Rearing Nuts which was increased to 2 kg per day as the calves grew. For the duration of the trial, the calves were offered water and silage *ad libitum* and were fed 2 kg concentrates (17% crude protein, 4% crude oil, 9.5% crude fibre, 7.5% crude ash, 0.28% magnesium, 0.28% sodium) (Calf Pride Rearing Nuts; John Thompson and Sons, Limited).

### Animal sampling

On days −1, 0, 6 and 7, relative to the challenge (day 0), a 9 ml K_3_ EDTA blood tube was collected, gently inverted several times and placed on ice (Fig. 1). Whole blood was analysed for haematological variables (white blood cell count, neutrophil percentage, lymphocyte percentage, monocyte percentage and eosinophil percentage) on an ABAXIS H5 haematology analyser (ABRAXIS Model: H5 S/N: 364372) immediately following collection. Daily, from the challenge to the day of slaughter, clinical signs (nasal discharge, ocular discharge, general demeanour, size of mandibular lymph nodes, presence of a cough, respiratory rate, respiratory character, mouth breathing, dyspnoea, presence of an expiratory grunt and rectal temperature) were recorded and scored by a veterinarian, who was blinded to the calves’ treatment status (BRSV challenged or control), (Fig. 1) using a modified version of the clinical scoring system described by Gershwin *et al*.^21^ (Supplementary Table S1). Using this scoring system, points were accrued for each abnormal clinical symptom and the total number of points corresponded with the severity of disease, such that higher clinical scores were associated with more severe BRD^21^.

On the day of the challenge, calves were either administered with 10^3.5^ TCID_50_/ml × 15 ml of BRSV inoculum (BRSV challenged calves in groups B and C) or mock challenged with 10 ml PBS (control calves in group A) by aerosol inhalation.

Animals were restrained in a cattle crush and the head was further restrained to prevent movement during nebulisation. Animals were nebulised with BRSV strain SVA 274/92^20^ or PBS using the Omron NE-U780 nebuliser (OMRON Model: NE-U780-E S/N: 20151200182AF) fitted with a veterinary face mask (GaleMed Model: VM-2 Animal Mask size 5 22mmlD Ref: 5635 Lot: 151019). Airflow and nebulisation volume settings were set to the maximum. BRSV inoculum (20 ml) was placed into the chamber and nebulised over 15 minutes. The remaining volume of inoculum was checked after 15 minutes and if less than 10 ml had been nebulised, nebulisation was continued for an additional 5–10 minutes.

On day 7 relative to the challenge, calves were euthanized by captive bolt. The lungs were scored for lesions by a veterinarian using an AFBI standard lung scoring system which assigns the percentages of lesions present on the total lung area and on component parts of the lungs (Supplementary Fig. S1).

The workspace and implements were thoroughly cleaned and disinfected with bleach and 75% ethanol and sprayed with RNaseZap for RNase inhibition, before tissue collection and between euthanisation of animals. Bronchial lymph node tissues were harvested immediately and flash frozen in liquid nitrogen, placed on dry ice and then transferred to a −80 °C freezer for storage.

### Statistical analysis of clinical and haematological data

**Figure 2:**
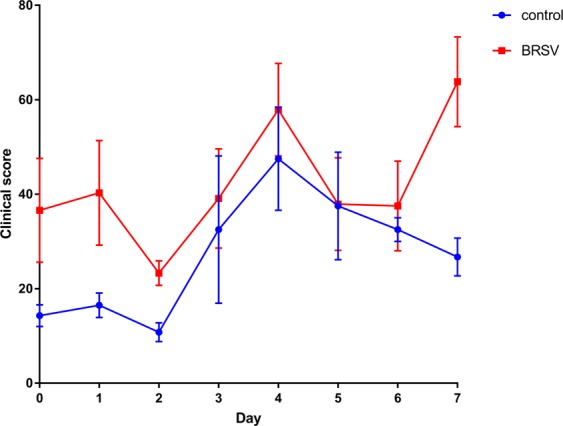
Clinical scores from the BRSV challenged (n = 12) and the control (n = 6) calves during the BRSV challenge study (means and their standard errors are presented). The day of the challenge is represented as day 0.

Clinical score and haematological variables (white blood cell number, neutrophil percentage, lymphocyte percentage, monocyte percentage and eosinophil percentage) were analysed using repeated measures mixed models (MIXED procedure of SAS v 9.4) where time-point defined the repeated measure. Data were first assessed for normality using PROC REG and PROC UNIVARIATE. All data were found to be normally distributed. Treatment (BRSV challenged or control), time-point or group (A, B or C) and their interactions were included as fixed effects. Calf was included as a random effect. A Tukey adjustment was used to correct for multiple testing. Means and their standard errors are presented in Figs 2 and S2 (graphs were generated in GraphPad Prism version 7.02). Lung scores were assessed for normality using PROC REG and PROC UNIVARIATE and analysed using a mixed model ANOVA (MIXED procedure of SAS v 9.4) with treatment (BRSV challenged or control) or group included as fixed effects. The presence or absence of lung lesions was analysed using a Fisher’s Exact test in SAS 9.4.

### RNA extraction

Total RNA (including miRNAs) was extracted using the Qiagen RNeasy Plus Universal Mini Kit (Qiagen LTD, Manchester, UK), according to the manufacturer’s instructions including Appendix C of the manufacturer’s protocol. The quantity of the extracted RNA was determined by measuring the absorbance at 260 nm with a Nanodrop spectrophotometer (NanoDrop technologies; Wilmington, DE, USA). The quality of the extracted RNA was examined with the Agilent 2100 Bioanalyser (Agilent Technologies Ireland Ltd; Dublin, Ireland) using the RNA 6000 Nano LabChip kit (Agilent Technologies Ireland Ltd; Dublin, Ireland). Samples had a mean RNA Integrity Number of 8.6 ± 0.31.

### RNA-Seq library preparation and sequencing

Extracted RNA was shipped frozen at −80 °C on dry ice to the University of Missouri’s DNA Core Facility for RNA-Seq library preparation using the TruSeq stranded mRNA Kit (Illumina, San Diego, California, USA) and high-throughput sequencing (75 bp paired-end) on an Illumina NextSeq 500. All sequence data produced in this study has been deposited to NCBI GEO repository and are available through the series accession number GSE131452.

### Alignment of sequence reads to the bovine reference genome and differential gene expression analysis

Adapter trimmed sequence reads in FASTQ format were assessed for quality using FastQC (version 0.11.7) (http://www.bioinformatics.babraham.ac.uk/projects/fastqc/). All reads passed the basic quality statistics. Reads were aligned to the UMD3.1 bovine reference genome and read counts were generated by converting aligned reads into counts per gene using the Spliced Transcripts Alignment to a Reference (STAR) aligner (*version* 2.5.2b)^22^.

Differential gene expression was determined using the R (R version 3.4.2 2017-09-28) Bioconductor package EdgeR (version 3.20.9)^23^ which accounts for biological and technical variation by modelling data as a negative binomial distribution. To remove lowly expressed genes, any genes with less than one count per million in at least three of the samples, were removed from the analysis. Data were normalised across libraries using the trimmed mean of M-values normalisation method^24^. The quantile-adjusted conditional maximum likelihood (qCML) common dispersion and the qCML tagwise dispersions were used to estimate dispersion. Exact tests were used for the detection of DEG between BRSV challenged and control calves. Genes with a Benjamini-Hochberg false discovery rate (FDR) of 10% and a fold change of ≥2 were considered differentially expressed.

### Pathway and gene ontology analysis

Statistically significant gene ontology terms, pathways and other gene function related terms were analysed from a ranked DEG list (DEG between BRSV challenged and control calves with an FDR of 10% and a fold change of >2, ranked by P-value) using g:Profiler version 2018-06-21^25^. Multiple testing correction was performed using the g:Profiler tailor-made algorithm g:SCS.

The DEG between BRSV challenged and control calves, with an FDR of 10% and a fold change of >2, were analysed for over-represented gene ontology terms/pathways and functional annotation clustering using the Database for Annotation, Visualization and Integrated Discovery (DAVID) (http://david.abcc.ncifcrf.gov/tools.jsp)^26^. The enrichment P-values for each annotation term were derived from a modified Fisher’s exact test called the EASE score. The group enrichment score for each functional annotation cluster was calculated from the geometric mean (in the -log scale) of all the enrichment P-values in each annotation term within each cluster and was used to determine the significance of results from the functional annotation clustering analysis. A group enrichment score threshold of 1.3 (EASE = 0.05) was applied.

To further examine over-represented pathways, cellular and molecular functions and predicted upstream regulators, the RNA-Seq data were analysed using the Ingenuity Pathway Analysis (IPA) (QIAGEN Inc., https://www.qiagenbioinformatics.com/products/ingenuitypathway-analysis), according to the manufacturer’s instructions^27^. Within IPA, Fisher’s exact test was used with the Benjamini-Hochberg correction for multiple testing for the identification of over-represented pathways and over-represented molecular and cellular functions with a FDR of 10%, from DEG between BRSV challenged and control calves, with an FDR of 10% and a fold change of >2. Additionally, IPA’s regulation Z-score algorithm, which predicts increases or decreases in functions based on directional changes in the DEG and expectations derived from the literature, was used to predict differences in the over-represented cellular and molecular functions. IPA software considered cellular and molecular functions with a regulation Z-score value of ≥2.0 to be significantly increased and cellular and molecular functions with a regulation Z-score value of ≤−2.0 to be significantly decreased.

### Comparison to the BRSV-associated RNA-Seq Data from Tizioto *et al*. (2015)

FASTQ files were downloaded from the National Center for Biotechnology Information Sequence Read Archive under accession number SRP052314 (SRR1952354, SRR1952355, SRR1952356, SRR1952357, SRR1952358, SRR1952359, SRR1952360)^18^. Alignment, gene counts, differential gene expression analysis, pathway and gene ontology analyses were performed identically to the analyses described for the current study. Pathway and gene ontology analyses were additionally performed on genes which were commonly differentially expressed in this study and that of Tizioto *et al*.^18^ with an FDR of 10% and a fold change of >2.

## Results

### Clinical score

Clinical score was affected by treatment (BRSV challenged 42.0 ± = 4.02 *versus* control 27.3 ± 5.68; P = 0.03) (Fig. 2). Time-point of clinical assessment also affected clinical scores (P < 0.0001) (Fig. 2) with days 4 (52.7 ± 7.42) and 7 (45.2 ± 7.42) being significantly greater than day 0 (25.5 ± 7.42). There was a tendency towards an interaction between time-point and treatment affecting clinical scores (P = 0.1), with the BRSV challenged calves having numerically greater clinical scores on day 7 versus day 2 (P < 0.1) and the control calves having significantly greater clinical scores on days 4 and 6 compared to day 2 (P < 0.05). However, there were no significant differences or tendencies between the BRSV challenged calves and the control calves at any of the time-points (P > 0.05). Furthermore, there was no effect of group on clinical scores (P > 0.05).

### Haematology variables

Control calves had greater (P < 0.0001) white blood cell counts (10.8 ± 0.47 × 10^9^/L) than the BRSV challenged calves (7.5 ± 0.33 × 10^9^/L) (Supplementary Fig. S2A). Time-point significantly influenced the white blood cell count (P < 0.006) which was lower on day 7 relative to day -1 (P < 0.05) (Supplementary Fig. S2A).

There were no effects of treatment (BRSV challenged *versus* control), time-point or an interaction between treatment and time-point for either neutrophil, monocyte or lymphocyte percentage (P > 0.05). While there were no effects of treatment, time-point or their interactions on eosinophil percentage (P > 0.05), there was an effect of group (A, B or C) (P = 0.02). BRSV challenged calves in group C had a greater percentage of eosinophils than the control calves in group A (P = 0.02) (BRSV challenged calves in group C; 7.3 ± 1.12%, BRSV challenged calves in group B; 4.3 ± 1.12%, control calves in group A; 2.3 ± 1.07%) (Supplementary Fig. S2B).

### Lung pathology

**Figure 3:**
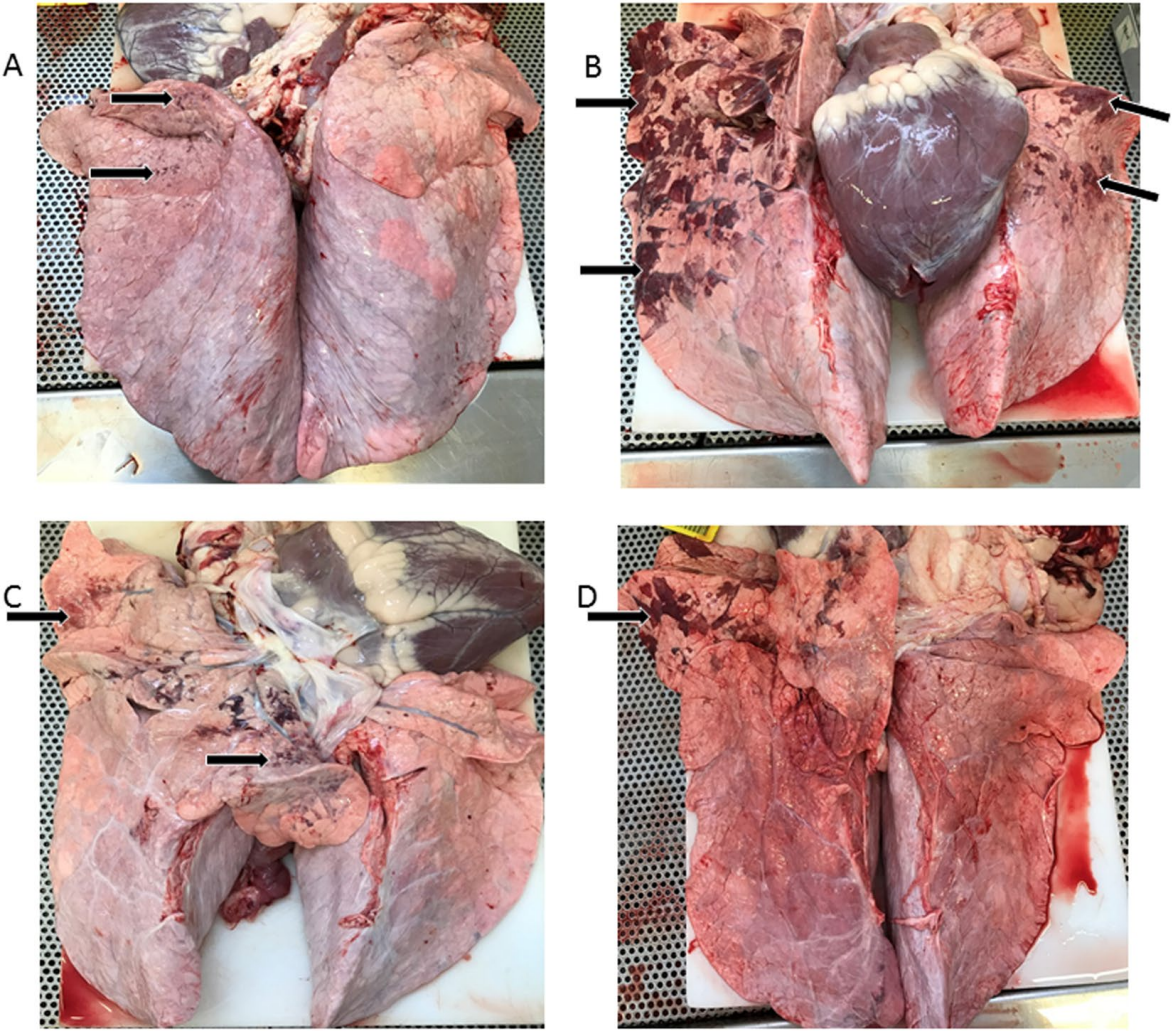
Photos of the lungs illustrating the pathologies from a subset of the BRSV challenged calves. Lesions are marked with black arrows. (**A**) = calf number 8, (**B**) = calf number 9, (**C**) = calf number 16, (**D**) = calf number 17.

Figure 3 shows the lung pathologies from a subset of the BRSV challenged calves. There were no differences in either the overall lung scores or the percentage of the right cranial lung lobe that was lesioned between the BRSV challenged calves and the controls (P > 0.05). This is likely due to the small percentages of lesioned tissues present on the lungs (BRSV challenged group average percentage of lesioned tissue overall was 1.34 ± 1.0% and the right cranial lobe 1.63 ± 0.8%, controls 0.08 ± 0.1% and 0.83 ± 0.8%) (Table 1).

**Table 1:** Lung scores (percentage of lungs with lesioned tissue) in control and BRSV challenged calves.

I.D.	Treatment	Group	Total Lung Score (%)	Lesioned Right Cranial Lobe (%)
1	control	A	0	0
2	control	A	0	0
3	control	A	0	0
4	control	A	0.46	5
5	control	A	0	0
6	control	A	0	0
7	BRSV	B	0	0
8	BRSV	B	0.22	0
9	BRSV	B	11.7	10
10	BRSV	B	0.06	1
11	BRSV	B	0.735	2
12	BRSV	B	0.03	0.5
13	BRSV	C	0.12	1
14	BRSV	C	0	0
15	BRSV	C	0	0
16	BRSV	C	0.71	2
17	BRSV	C	1.3	2
18	BRSV	C	1.19	1

However, overall, only one control calf exhibited a lesioned lung, while 5 out of the 6 calves in BRSV challenge group B and 4 out of the 6 calves in BRSV challenge group C had lung lesions (Table 1). The BRSV challenged calves had a higher probability of having a lesioned lung compared with the control calves (P = 0.04).

### Sequence alignment

An average of 53,878,694 (2 × 75 bp) paired-end reads (i.e. 26,939,347 sequenced fragments) were generated for each sample (Supplementary Table S2). Overall, 7.2% of the sequence reads failed to map to the genome and 89.6% of sequence reads mapped uniquely to the genome. On average, 57.3% of the total number of sequence reads were assigned to a gene by STAR, 0.4% were ambiguous and 31.9% of the sequence reads aligned to no feature (Supplementary Table S2).

### Differential gene expression and functional annotation

**Figure 4:**
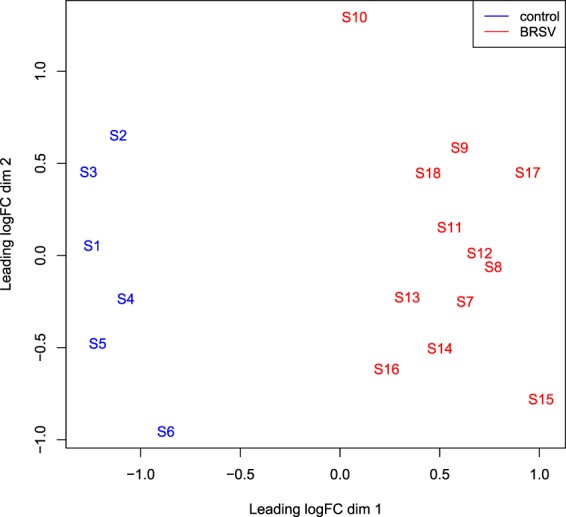
An MDS plot generated in EdgeR illustrating the similarity of the samples based on the first two principal components of gene expression covariance matrix among individuals. Samples from BRSV challenged calves are coloured in red and samples from control calves are coloured in blue. The numbers (1–18) refer to the calf ID and the letter S in front of the numbers refers to the word “sample”.

Using Edge-R, a multi-dimensional scaling (MDS) plot was produced which allowed an examination of the similarity of the samples based on global gene expression when samples were plotted using their first two principal components. The MDS plot indicated no differences between individuals in BRSV challenge groups B and C, but a clear separation between the BRSV challenged and the control calves (Fig. 4).

There were 934 DEG (p < 0.05, FDR < 0.1, fold change >2) between the BRSV challenged and control calves (Supplementary Table S3). There were two DEG between the calves in BRSV challenge group B and BRSV challenge group C (*PPP1R1B* and *LYNX1*).

**Figure 5:**
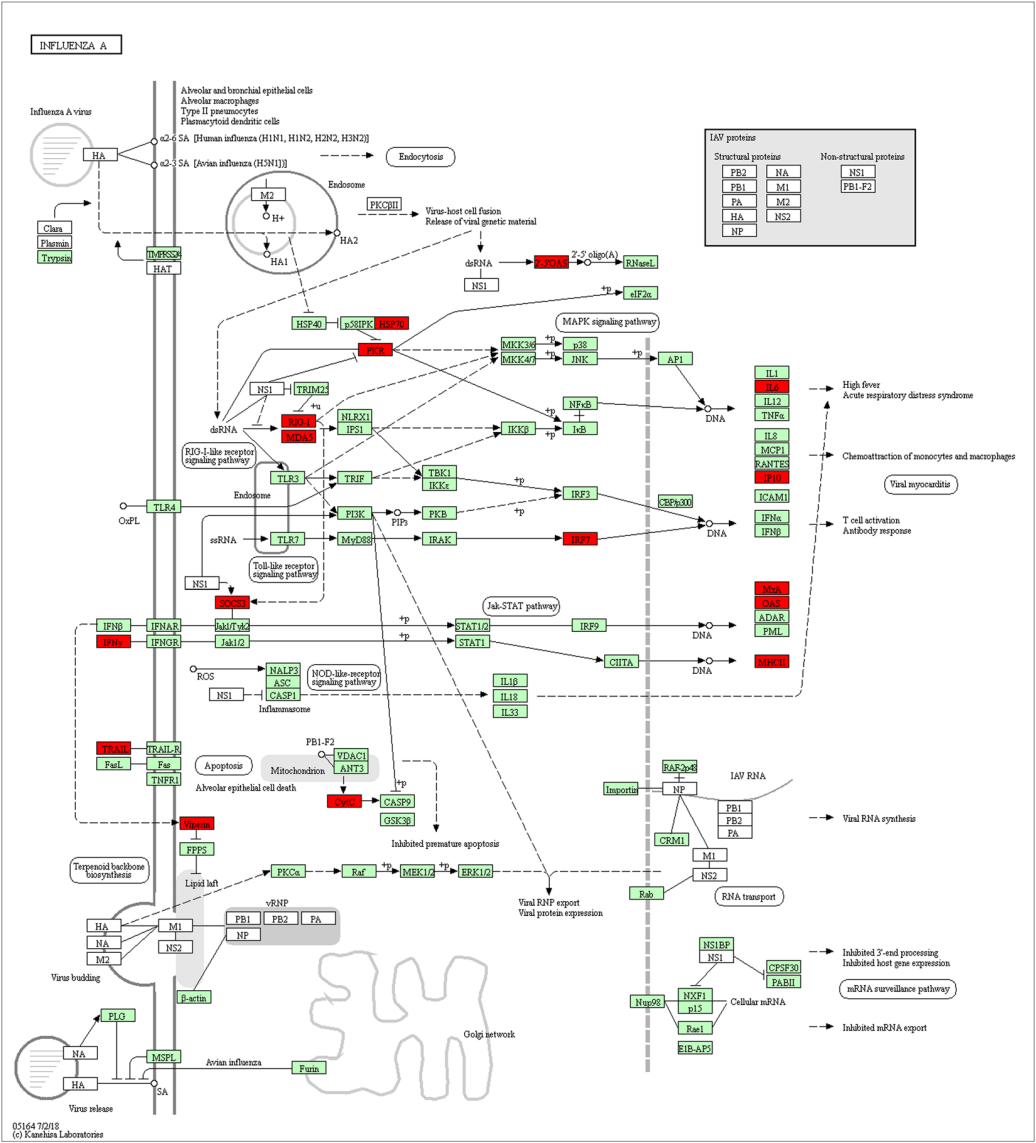
Genes up-regulated (red) in BRSV challenged calves (n = 12) in the KEGG:05164 Influenza A pathway^28^ (https://www.genome.jp/kegg/tool/map_pathway2.html).

From the g:Profiler analysis, there were 133 over-represented biological process Gene Ontology terms, 26 over-represented cellular component Gene Ontology terms, 23 over-represented molecular function Gene Ontology terms, 17 molecular function KEGG pathways and 20 over-represented Reactome pathways (P_corrected_ <0.05) (Supplementary Table S4). The 12 most significant over-represented biological process Gene Ontology terms were related to the immune response (Table 2). The over-represented KEGG pathways were also associated with immune response and diseases and included KEGG:05164 Influenza A (Fig. 5)^28^. This pathway contained 19 up-regulated and two down-regulated genes (Supplementary Table S4, Fig. 5).

**Table 2:** The 12 most significant over-represented biological process Gene Ontology terms among the genes which were differentially expressed between BRSV challenged and control calves.

Term ID	Term name	P-value
GO:0006952	defense response	6.59E-17
GO:0006955	immune response	3.75E-16
GO:0002376	immune system process	4.07E-16
GO:0002252	immune effector process	4.96E-14
GO:0009605	response to external stimulus	2.68E-12
GO:0006950	response to stress	2.43E-11
GO:0009607	response to biotic stimulus	1.19E-10
GO:0098542	defense response to other organism	3.29E-10
GO:0051707	response to other organism	4.78E-10
GO:0043207	response to external biotic stimulus	6.05E-10
GO:0002377	immunoglobulin production	3.17E-09
GO:0009615	response to virus	2.99E-08

There were 77 over-represented ontology terms in the DAVID analysis (p < 0.05, Benjamini-Hochberg FDR <0.1) (Supplementary Table S5). These were associated with the immune response, glycoproteins and secreted peptides. There were 28 enriched functional annotation clusters in the DAVID analysis (group enrichment score >1.3) (Supplementary Table S6). The enriched clusters contained terms relating to signal peptides, immunity, immunoglobulin, nucleotide-binding, thrombospondin, endoplasmic reticulum, double-stranded RNA binding, viral diseases, inflammatory responses, lectins, chemokine activity, extracellular matrix structure, cell division, calcium binding and the complement pathway.

**Figure 6:**
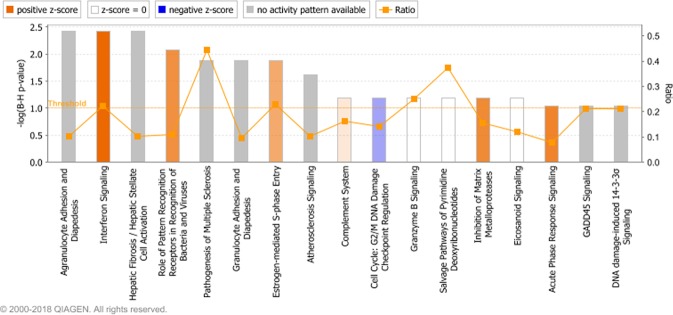
The enriched canonical pathways found in the IPA analysis^27^ (P < 0.05, FDR <0.1). The pathways are shown on the x-axis and the –Log Benjamini-Hochberg adjusted p values are displayed on the y-axis. The threshold is set to 1 which equals a Benjamini-Hochberg adjusted p value of 0.1. The orange line representing the ratio corresponds to the ratio of the number of differentially expressed genes that map to the pathway divided by the total number of genes that map to the same pathway. Pathways with a positive z-score are predicted by IPA to have increased activity and pathways with a negative z-score are predicted to have decreased activity.

Ingenuity pathway analysis showed that seventeen canonical pathways were enriched (P < 0.05, FDR <0.1) (Supplementary Table S7, Fig. 6). Four of these pathways were predicted to be upregulated (Interferon Signaling, Role of Pattern Recognition Receptors in Recognition of Bacteria and Viruses, Inhibition of Matrix Metalloproteases and Acute Phase Response Signaling) (Z-score >2) (Supplementary Table S7, Fig. 6).

Thirty-one enriched biological functions were predicted to be increased in BRSV challenged animals compared to controls (Z-score >2) (Supplementary Table S8). Their functions were related to the recruitment, activation and death of leukocytes.

There were 256 up-stream regulators that were predicted by IPA to be activated (Z-score ≥2) (Supplementary Table S9). Nine of these had an observed increase in fold change (*IFNG, IRF7, DDX58, CGAS, OSM, IL27, EIF2AK2, XBP1* and *TBX21*). There were 84 up-stream regulators that were predicted to be inhibited by IPA (Z-score ≤2) (Supplementary Table S9), but none of these were found to have a decreased expression in the BRSV challenged calves.

### Differential gene expression and functional annotation of RNA-Seq data in comparison to Tizioto *et al*. (2015) study

An average of 106,159,694 paired-end reads (2 × 50 bp) (i.e. 53,079,847 sequenced fragments) were obtained from each BRSV challenged/control bronchial lymph node sample downloaded from the National Center for Biotechnology Information Sequence Read Archive under accession number SRP052314 (Supplementary Table S2)^18^. Overall, 11.7% of sequence reads failed to map to the genome and 82.7% of reads mapped uniquely to the genome, using the STAR aligner^22^. On average, 28.0% of the total number of sequence reads were assigned to a gene by STAR, 0.1% were ambiguous and 54.6% of reads aligned to no feature (Supplementary Table S2).

**Figure 7:**
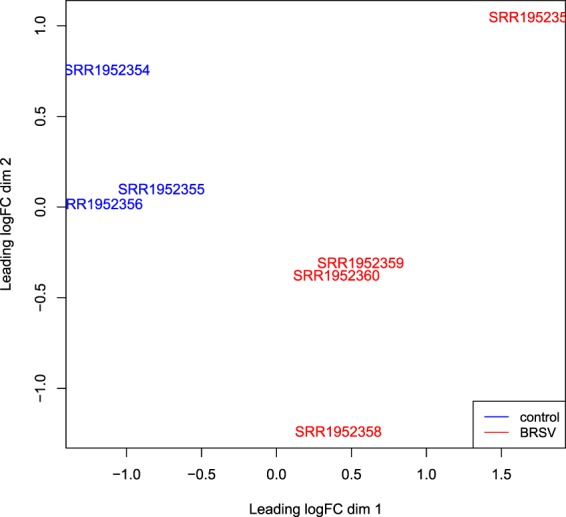
An MDS plot generated in EdgeR illustrating the similarity of the Tizioto *et al*.^18^ BRSV challenged (n = 4) and control (n = 3) bronchial lymph node samples based on log_2_ fold gene expression changes. Samples from BRSV challenged calves are coloured in red and samples from control calves are coloured in blue. The sample names are the accession numbers of the samples.

**Figure 8:**
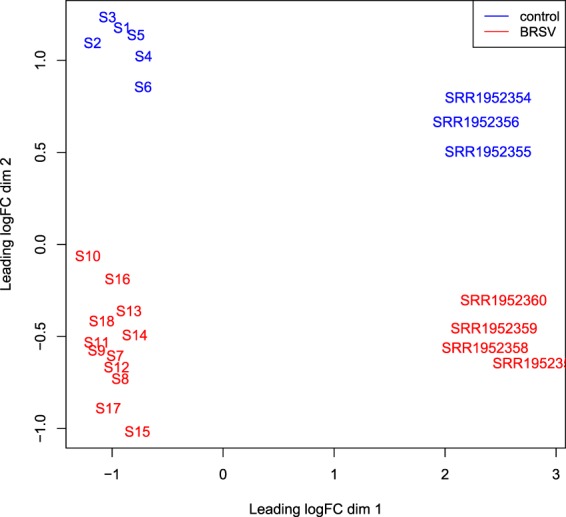
An MDS plot (based on log_2_ fold gene expression changes) generated in EdgeR illustrating the similarity between the BRSV challenged (n = 12) and control (n = 6) bronchial lymph node samples from the present study and from the Tizioto *et al*.^18^ study.

An MDS plot illustrating the similarity of the Tizioto *et al*.^18^ BRSV challenged and control bronchial lymph node samples based on log_2_ fold gene expression changes was produced in EdgeR (Fig. 7). For illustration of the similarities between the samples in the present study and those from the Tizioto *et al*.^18^ study, an MDS plot was produced in EdgeR based on log_2_ fold gene expression changes among all samples (Fig. 8).

One thousand and thirteen genes from the study of Tizioto *et al*.^18^ were differentially expressed between BRSV challenged and control calves (Supplementary Table S10). From the g:Profiler analysis, there were 198 over-represented biological process Gene Ontology terms, 19 over-represented cellular component Gene Ontology terms, 16 over-represented molecular function Gene Ontology terms, 18 over-represented KEGG pathways, 38 over-represented Human Phenotype Ontology terms, 1 over-represented CORUM term and 30 over-represented Reactome pathways (P_corrected_ <0.05) (Supplementary Table S11). The over-represented biological process Gene Ontology terms were related to the immune response. The over-represented KEGG pathways were also associated with immune responses and diseases and included KEGG:05164 Influenza A and KEGG:04060 Cytokine-cytokine receptor interaction.

There were 354 DEG in common between the present study and the Tizioto *et al*.^18^ study (BRSV challenged and control bronchial lymph node samples) (Supplementary Table S12). From the g:Profiler analysis, there were 81 over-represented biological process Gene Ontology terms, 18 over-represented cellular component Gene Ontology terms, 13 over-represented molecular function Gene Ontology terms, 16 over-represented KEGG pathways, 12 over-represented Human Phenotype Ontology terms, 1 over-represented CORUM term and 20 over-represented Reactome pathways (P_corrected_ <0.05) among the DEG in common between the two data sets (Supplementary Table S13).

**Figure 9:**
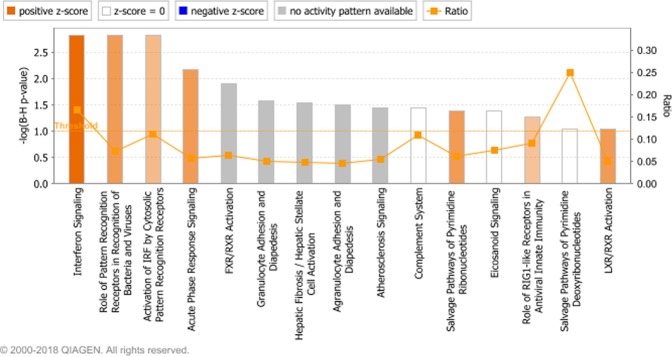
The enriched canonical pathways in the IPA analysis^27^ among DEG in common between the present study and the Tizioto *et al*.^18^ study (P < 0.05, FDR <0.1). The pathways are shown on the x-axis and the –Log Benjamini-Hochberg adjusted p values are displayed on the y-axis. The threshold is set to 1 which equals a Benjamini-Hochberg adjusted p value of 0.1. The orange line representing the ratio corresponds to the ratio of the number of differentially expressed genes that map to the pathway divided by the total number of genes that map to the same pathway. Pathways with a positive z-score are predicted by IPA to have increased activity and pathways with a negative z-score are predicted to have decreased activity.

Pathway analysis in IPA revealed that 15 pathways were over-represented among the DEG in common between the present study and the Tizioto *et al*.^18^ study (Fig. 9, Supplementary Table S14). The most significant of the over-represented pathways, interferon signaling and the role of pattern recognition receptors in recognition of bacteria and viruses, were also among the most significant over-represented pathways in the IPA pathway analysis of the DEG solely found in this study.

Fourteen enriched biological functions, among DEG in common between the present study and the Tizioto *et al*.^18^ study, were predicted to be increased in BRSV challenged animals compared to controls (Z-score >2) (Supplementary Table S15). Functions were related to the antimicrobial and antiviral immune response. Twelve enriched biological functions, among DEG in common between the present study and the Tizioto *et al*.^18^ study, were predicted to be decreased in BRSV challenged animals compared to controls (Z-score >2) (Supplementary Table S15). These functions were associated with viral replication and morbidity.

## Discussion

This is the first study to examine the host response at the transcriptional level in artificially-reared Holstein-Friesian calves to a challenge with BRSV. Previous studies have examined the bronchial lymph node pathological response to a BRSV challenge in Angus-Hereford crossbred beef calves^18,19,21^. However, immune responses, particularly at the level of transcription, have been demonstrated to be affected by breed^29–31^. This may be the reason that the majority of the variation observed in the MDS plot (Fig. 8) was due to study (the present study *versus* Tizioto *et al*.^18^), while treatment (BRSV challenged *versus* control) bronchial lymph node gene expression was the secondary source of variation. However, the different sequencing platforms used (NextSeq 500 *versus* HiSeq 2000), the varying length and depth of reads generated, the distinct BRSV viral strains used in the inoculum and the alternative husbandry and management practices carried out in the studies’ diverse geographical locations, may have also influenced the alterations in gene expression between the two studies. Despite the many sources of variation existing between the two studies, 38% of the DEG in this study were commonly differentially expressed in the RNA-Seq analysis of the BRSV challenged and control bronchial lymph node samples from the study of Tizioto *et al*.^18^. Furthermore, the majority of enriched pathways found in the two studies were in common. Therefore, BRSV appears to elicit many conserved alterations in gene expression.

In the present study, calves were challenged with a similar titre of BRSV that has consistently and successfully been used to establish infection in previous BRSV challenge studies^18,21,32^. Calves were euthanised 7 days post challenge as this is the time-point when the acute phase response has been observed to peak^33^ and when live BRSV and its antigen titres begin to decrease^34^. Bronchial lymph node tissues were harvested and RNA-Seq analysis was performed on these tissues to determine the gene expression changes induced by BRSV infection as these lymph nodes drain the lungs and are the site of antigen presentation and the initiation of the primary immune response^35,36^.

Infection with BRSV can be subclinical, present only in the upper respiratory tract or it can affect both the upper and lower parts of the respiratory tract^15^. BRSV mainly infects and produces clinical signs in calves less than one year of age but is usually more severe in young calves aged between one and three months^32^. It is common for paramyxoviruses, including BRSV, to be attenuated by passage through *in vitro* culture, and this can result in minimal clinical symptoms of respiratory disease^37^. Therefore, the age of the calves (approximately 5 months of age) and the effects of BRSV attenuation in culture may have influenced the primarily sub-clinical response to BRSV challenge observed in the present study. The calves in this study had substantially lower clinical scores than the calves challenged with BRSV in the Tizioto *et al*.^18,21^ study. This may have been due to the possible differing initial BRSV specific MDA levels of the calves, the diverse breeds of the calves or the distinct strains of BRSV inoculum used for the challenge. Furthermore, there were no changes in neutrophil, lymphocyte or monocyte percentages due to the BRSV challenge in this study. While control calves did have a higher white blood cell count compared with BRSV challenged calves throughout the study, the white blood cell numbers were within the normal range for healthy cattle^38^ and no increase in white blood cell count was observed following virus inoculation, in BRSV challenged calves.

Interestingly, despite these BRSV challenged calves only showing a modest clinical and haematological response to infection, and having only a small proportion of their lungs lesioned, the virus induced vast changes in bronchial lymph node gene expression levels. Therefore, measuring host gene expression levels may prove to be a more accurate way of diagnosing BRD than by the observation of clinical signs of the disease. This would be hugely beneficial to the global agricultural industry as sub-clinical BRD which remains undiagnosed is almost as prevalent in healthy cattle as BRD is among cattle which are diagnosed with respiratory disease and treated for BRD^39^. Although it would clearly not be possible to measure host gene expression in bronchial lymph node tissue in live calves if a small set of diagnostic genes are also perturbed in expression in white blood cells or nasal fluid, these more accessible media could be used as “liquid biopsies” for the future diagnosis of subclinical respiratory infections. Indeed increased expression of *IFNG* and *IL6* have been observed along with clinical signs during an initial BRSV challenge and the increased expression of *IFNG* in the peripheral blood mononuclear cells of young calves with an absence of clinical symptoms has also been observed in a reinfection with BRSV^34^.

The majority of the alterations in bronchial lymph node gene expression due to BRSV challenge detected in the present study were associated with the immune response. The most highly upregulated gene was *GZMB*, which was upregulated sixty-seven fold. This gene was also the most up-regulated in response to a BRSV challenge in bronchial lymph node in US calves^18^ and the encoded protein has been shown to be significantly increased in CD8+ and CD4+ T cells and natural killer cells, in tracheal aspirates from children infected with respiratory syncytial virus (RSV)^40^. Furthermore, *GZMB* was found to be a member of several enriched ontologies and pathways including “defense response”, “immune response”, “granzyme-mediated apoptotic signaling pathway” and “granzyme B signaling”, in the present study. The serine protease protein encoded by this gene is one of the major components of the lytic granules which are released by cytotoxic T cells and natural killer cells to destroy virally infected cells^41^. It also plays a role in degradation of the extracellular matrix and induction of pro-inflammatory cytokine responses^42^. Furthermore, granzyme B has recently been found to be utilised by T regulatory cells to control effector B and T cell populations, thereby regulating the inflammatory response and reducing the severity of RSV disease in mice^43^. Consequently, it is likely to play an important role in defence to BRSV infection and alterations to this gene may be critically important to BRD resistance and susceptibility. Indeed, it has been shown that polymorphisms in murine *GZMB* can reduce the ability of CD8+ T cells to kill virally infected target cells^44^. It has also been postulated that that *GZMB*-expressing T regulatory cells could be used as a therapeutic strategy in human viral lung infections, due to their ability to dampen inflammation without elevating viral load^43^. However, a study of mice experimentally infected with *Klebsiella pneumoniae* found that while *GZMB* knockout mice had higher levels of lung inflammation at earlier stages in the disease, there were minimal differences in distant organ injury or survival between the knockout and wild type mice^45^. However, *Klebsiella pneumoniae* is caused by a bacterium rather than a virus and as *GZMB* is mainly involved in the cell mediated cytotoxic immune response, it may be more effective in the elimination of viral infections and intracellular bacteria.

Pathways and gene ontologies involved in the innate immune response, induction of cytokines and chemokines, humoral and cell mediated immunity were over-represented among the DEG. The KEGG pathway for Influenza A was enriched for DEG and this pathway contains upregulated cytokines responsible for the induction of the febrile response, mitochondrial genes involved in programmed cell death and interferon stimulated genes which induce cytotoxic T cell responses. Tizioto *et al*.^18^ also observed an enrichment of similar pathways and the DEG found in common in both studies were members of the most significantly enriched pathways from this study, including interferon signaling and role of pattern recognition receptors in recognition of bacteria and viruses. Furthermore, the induction of pro-inflammatory chemokines and cytokines in calves due to BRSV infection has been reported in pneumonic lung lesions^46^, bronchoalveolar lung fluid^47^ and bovine respiratory/turbinate epithelial cells^48^. Therefore, these immune response associated genes seem to play an important role in the bovine host response to BRSV infection. This is expected as pathogen recognition, induction of inflammatory cytokines and chemokines, and the initiation of the innate immune response are vital to control the virus upon entry. Subsequent elimination of the virus requires the development of CD8 cytotoxic T cells, which is facilitated by the production of *IFNG* and interferon stimulated genes such as *DDX58, IFI44, IFIH1, IFIT1, IFIT2, IFIT3, IFIT5, ISG15, ISG20, MX1, MX2, OAS1, EIF2AK2, RTP4, RSAD2, OAS2* and *OSM*, which were upregulated in the present study^49^.

In agreement with Tizioto *et al*.^18^, we also predicted neurodegenerative diseases and functions including “progressive motor neuropathy”, “progressive neurological disorder”, “inflammatory demyelinating disease” and “multiple sclerosis” to be increased in the BRSV challenged calves. These findings are interesting as human RSV has been observed as being capable of infecting murine lung neuronal cells^50^, and also of infecting the human central nervous system and inducing neurological symptoms^51^.

BRSV modulates the immune response towards a T helper 2 (Th2) type response, with increased expression of Th2 promoting cytokines and increased concentrations of BRSV specific IgE antibodies in lymph fluid^48^. Despite enriched ontology terms including “B cell activation” and “immunoglobulin mediated immune response”, we found no evidence for an increase in the transcription of either *IL4* or *IL13* which initiate the Th2 bias^52^. This is consistent with the results of Grell *et al*.^34^, who did not detect an increase in *IL4* gene expression following a BRSV challenge. However, although there was a 2 fold increase in the expression of *IL12RB2*, a transmembrane protein subunit of the interleukin 12 receptor complex, there was no increase in the expression of *IL12*, which is necessary to induce Th1 cell proliferation responses^48^, which is consistent with the expected Th2 bias for BRSV.

Lipopolysaccharide (LPS) was a predicted upstream regulator in this study and that of Tizioto *et al*.^18^. Furthermore, *LBP* was the second greatest up-regulated gene in response to the BRSV challenge infection in this study (fold change = 52). This is likely because the fusion protein from BRSV interacts with TLR4 to initiate the innate immune response in a manner that is similar to LPS^48^. Tizioto *et al*.^18^ suggested that LPS has the potential to be used in new treatments aiming to prevent the occurrence of BRD as it induces a cascade of both anti-viral and anti-bacterial genes. Other predicted upstream regulators in the present study include *IFNG* and *IRF7*, which play roles in IFN antiviral innate immune responses^53^.

BRSV is closely related to human RSV, and induces similar pathologies^15,37^ and alterations in host gene expression. Genes involved in the type 1 interferon antiviral response, including *BP1, RSAD2, MX2, MX1, ISG15, IRF7, IFITM3, IFIT3, IFIT2, IFIT1, IFIH1, IFI44*, which were upregulated in the lungs of C57BL/6 mice, in response to infection with RSV^54^, were also up-regulated in the calves challenged with BRSV in the present study. Therefore, the gene expression changes induced by BRSV infection detected in this study may provide an insight into the human bronchial lymph node transcriptional response to RSV. Future studies will also elucidate the bovine host micro-RNA gene expression responses and determine the regulatory regions controlling these transcriptional alterations due to infection with BRSV, in bronchial lymph node, and in other important respiratory tissues including lesioned and healthy lung tissue, mediastinal and retropharyngeal lymph nodes, and the pharyngeal tonsil. The DEG identified in this study, particularly those also observed to be DEG in the study of Tizioto *et al*.^18^, and therefore involved in the bovine host response to BRSV infection along with the regulatory regions controlling transcriptional changes in these genes may be interrogated in future large scale studies for the identification of variants which may be associated with resistance to BRSV infection. These variants could be included on genotyping assays routinely used in the international beef and dairy cattle industries for use in breeding programmes targeted on the generation of healthier, more robust cattle with a greater potential to resist BRSV infection and subsequent BRD.

## Supplementary information

Supplementary Figure S1, Supplementary Figure S2, Supplementary Table S1

Supplementary Table S2.

Supplementary Table S3.

Supplementary Table S4.

Supplementary Table S5.

Supplementary Table S6.

Supplementary Table S7.

Supplementary Table S8.

Supplementary Table S9.

Supplementary Table S10.

Supplementary Table S11.

Supplementary Table S12.

Supplementary Table S13.

Supplementary Table 14.

Supplementary Table S15.

## Data Availability

All sequence data produced in this study has been deposited to NCBI GEO repository and are available through series Accession Number GSE131452.
