# Supplementary material for: Experimental challenge with bovine respiratory syncytial virus in dairy calves: bronchial lymph node transcriptome response
Source: Sci Rep. 2019 Oct 14;9:14736. doi: 10.1038/s41598-019-51094-z (PMC6791843; doi:10.1038/s41598-019-51094-z)
Supplement: Supplementary file 1 — Supplementary Figure S1, Supplementary Figure S2, Supplementary Table S1 [file 41598_2019_51094_MOESM1_ESM.pdf]

Experimental challenge with bovine respiratory syncytial virus in dairy calves: bronchial lymph node transcriptome response

Dayle Johnston, Bernadette Earley, Matthew S. McCabe, Ken Lemon, Catherine Duffy, Michael McMenamy, S. Louise Cosby, JaeWoo Kim, Gordon Blackshields, Jeremy F. Taylor and Sinead M. Waters

Supplementary Figure S1. Scoring of lung lesions.

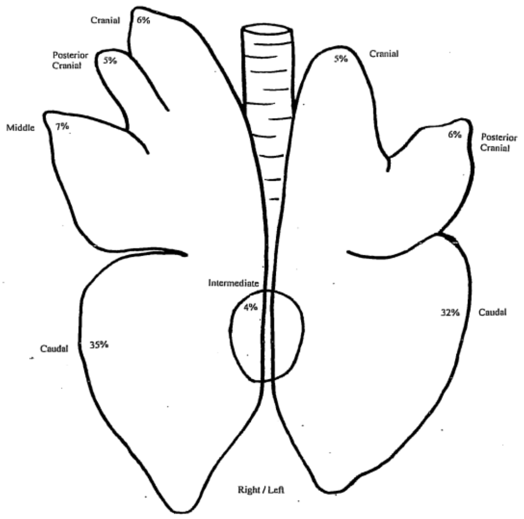

Lung lobe	A: Percent area of lobe with lesion	B: Lobe area (% of total)	(A x B)/100
Right Cranial		6	
Right Posterior Cranial		5	
Right Middle		7	
Right Caudal		35	
Intermediate		4	
Left Cranial		5	
Left Posterior Cranial		6	
Left Caudal		32	
		Total lung score out of 100%	

Furthermore, the pneumonic lesions will be defined as:

i. Acute bronchopneumonia
ii. Subacute fibrinopurulent bronchopneumonia
iii. Percentage of pneumonic tissue
iv. Consolidation
v. Interstitial oedema
vi. Abscesses
vii. Necrotic foci
viii. Haemorrhage
ix. Other pneumonic lesions (pleuritis and emphysema)

Supplementary Figure S2. Haematology counts.

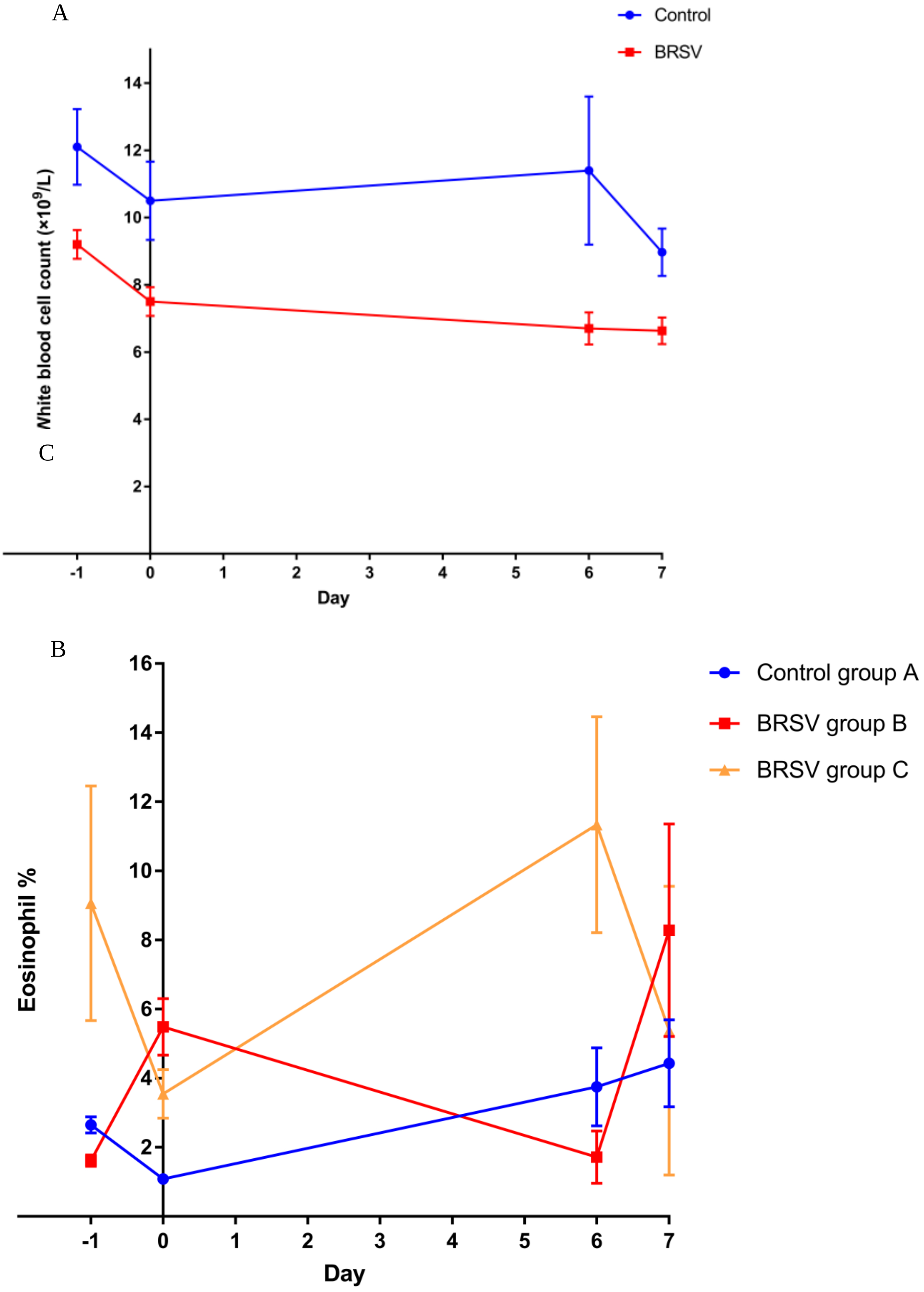

Supplementary Figure S2. Haematology cell counts. A) White blood cells ($\times 10^9$/L), and B) Eosinophil (%) in BRSV challenged (groups B and C) and control calves (group A).

Supplementary Table S1. Respiratory clinical signs scoring.

Parameter Evaluated	Score Scale
Rectal temperature	(T-39.5°C) X 40
Nasal discharge (each nostril scored separately)	5 (serous), 10 (seromucoid), 15 (mucoid), 20 (mucopurulent)
Ocular discharge (each eye scored separately)	5 (serous), 10 (seromucoid), 15 (mucoid), 20 (mucopurulent)
Spontaneous cough 0 (no), 40 (yes)	0 (no), 40 (yes)
Induced cough 0 (no), 40 (yes)	0 (no), 40 (yes)
Respiratory Rate (RR)–breaths/minute	RR-40
Subcutaneous emphysema 0 (no), 100 (yes)	0 (no), 100 (yes)
Mandibular lymph nodes 0 (normal), 50 (enlarged)	0 (normal), 50 (enlarged)
Lung sounds (harsh or quiet; each lung scored separately)	0 (normal), 15 (mild/moderate), 30 (severe)
Crackles	0 (no), 80 (yes)
Wheezes	0 (no), 60 (yes)
Respiratory character	0 (normal), 30 (shallow or deep)
Apneustic breathing	0 (no), 30 (yes)
Mouth breathing	0 (no), 50 (yes)
Dyspnea	0 (no), 75 (yes)
Expiratory grunt	0 (no), 60 (yes)
Biphasic expiration	0 (no), 60 (yes)
General clinical condition	0 (**Normal:** Bright, alert, bright eyes, erect ears, attentive, licks nostrils frequently and stays with the group); 30 (**Subdued:** Ears drop slightly, licks nostrils occasionally, tries to stay with the group); 70 (**Depressed:** Walks slowly, lethargic, stands alone for prolonged periods, sometimes stands with head down); 100 (**Unresponsive:** Uninterested in environment, very lethargic, apathetic, stands with head down most of the time, often in sternal decumbency, reluctant to stand up. Euthanasia for ethical reasons.
